# Supplementary material for: Sex Differences in Diabetes- and TGF-β1-Induced Renal Damage
Source: Cells. 2020 Oct 3;9(10):2236. doi: 10.3390/cells9102236 (PMC7600610; doi:10.3390/cells9102236)
Supplement: Supplementary file 1 [file cells-09-02236-s001.pdf]

cells

Supplementary Material and Methods:

Chromosome Preparation and Sex Determination

In order to characterize the TKPTS cells, a chromosomal preparation was carried out. The cells were cultured in 75 cm2 flasks until they reach a confluence of nearly 80% and had enough proliferating, round cells. To arrest the cell cycle in mitosis of the metaphase, 50 µl colcemid (10 µl/ml, Sigma Aldrich, Merck, Darmstadt, Germany) was transferred to the cells, followed by an incubation time of 3 h. After cell harvesting, they were centrifuged (1500 rpm, 5 min) and only 0.5 ml of the supernatant was used for a gentle resuspension of the cell pellet, so that the cells could be treated carefully and successively with 4 ml of preheated (37 °C) hypotonic solution. Following this, the cells were incubated (20–25 min) and centrifuged (1500 rpm, 5 min) once again. Then, 4 ml ice-cold (4 °C) fixative (methanol/acetic acid (Roth, Karlsruhe, Germany) in a ratio 3:1) was carefully added to the cells, while the cells were mixed periodically. This step was repeated twice in order to wash the cells. In a terminal step, the swollen and fixed cell suspension was plated onto cold, wet object slides, causing the burst of the cell membrane. In the end, interphase nuclei and metaphase chromosomes were exposed. The Giemsa bands (G-bands) were obtained by digesting the chromosomes with Trypsin solution (Trypsin 0.05%, 1:250, 50 mg; and Dulbecco´s phosphate-buffered saline-calcium- and magnesium-free medium (Dulbecco´s PBS-CMF), 100 ml) for 5 min, rinsing them briefly in cold (2–5 °C) Dulbecco´s PBS-CMF medium, staining them in Giemsa solution (5%, 5–6 min), rinsing them once again in Dulbecco´s PBS-CMF medium and allowing them to dry. Fluorescence in situ hybridization (FISH) was performed as previously reported [82]. As probes, a homemade glass needle microdissection derived whole chromosome painting (wcp) probe for the murine Y-chromosome was used [83].

82. Guja, K.; Liehr, T.; Rincic, M.; Kosyakova, N.; Hussein Azawi, S.S. Molecular Cytogenetic Characterization Identified the Murine B-Cell Lymphoma Cell Line A-20 as a Model for Sporadic Burkitt's Lymphoma. J Histochem Cytochem **2017**, 65, 669–677.

83. Kosyakova, N.; Trifonov, V.; Romanenko, S.; Mkrtchyan, H.; Graphodatsky, A.; Liehr, T. Murine multicolor banding. Tsitologiia **2013**, 55, 259-–60.

Supplementary Tables and Figures:

Table S1: Clinical/laboratory data in male and female mice with a non-diabetic or diabetic phenotype.

	**non-diabetic male**	**non-diabetic female**	**diabetic male**	**diabetic female**	**Sex Sign. (*P*)**	**Diabetes Sign. (*P*)**	**Sex*Diabetes Sign. (*P*)**
body weight (g)	25.6 ± 0.6	19.0 ± 0.8	44.8 ± 1.2	51.6 ± 1.5	0.935	**0.000*** **	**0.000*** **
blood glucose (mmol/l)	6.7 ± 0.4	6.3 ± 0.6	8.2 ± 1.1	8.4 ± 0.8	0.886	**0.027***	0.717
white blood cells (10^9/l)	3.1 ± 0.7	4.0 ± 0.7	3.7 ± 0.7	3.6 ± 0.4	0.577	0.851	0.467
red blood cells (10^{12}/l)	9.7 ± 0 .2	9.8 ± 0.2	10.4 ± 0.2	9.7 ± 0.2	**0.073**$^{(*)}$	0.219	**0.086**$^{(*)}$
platelets (10^9/l)	1297 ± 160	935 ± 68	938 ± 66	807 ± 60	**0.024***	**0.026***	0.272
hemoglobin (mmol/l)	13.9 ± 0.2	13.9 ± 0.2	15.4 ± 0.3	15.0 ± 0.2	0.338	**0.000*** **	0.307
hematocrit (l/l)	47.7 ± 0.7	47.6 ± 0.7	52.8 ± 1.1	49.7 ± 0.7	**0.057**$^{(*)}$	**0.000*** **	**0.079**$^{(*)}$
MCV (μm^3)	48.9 ± 0.4	48.8 ± 0.2	51.0 ± 0.5	51.2 ± 0.4	0.919	**0.000*** **	0.705
MCH (fmol)	0.88 ± 0.01	0.88 ± 0.01	0.92 ± 0.01	0.95 ± 0.01	**0.074**$^{(*)}$	**0.000*** **	0.118
MCHC (mmol/l)	18.0 ± 0.1	18.1 ± 0.1	18.2 ± 0.2	18.7 ± 0.1	**0.003** **	**0.001*** **	**0.019** **
urine output (μl)	1355 ± 248	481 ± 138	1525 ± 214	1286 ± 200	**0.011***	**0.025***	0.135

MCH, mean corpuscular hemoglobin. MCHC, mean corpuscular hemoglobin concentration. MCV, mean corpuscular volume. Values are the means ± SEM.

Number of mice per group (non-diabetic male/non-diabetic female/diabetic male/diabetic female = 10/8/8/7).

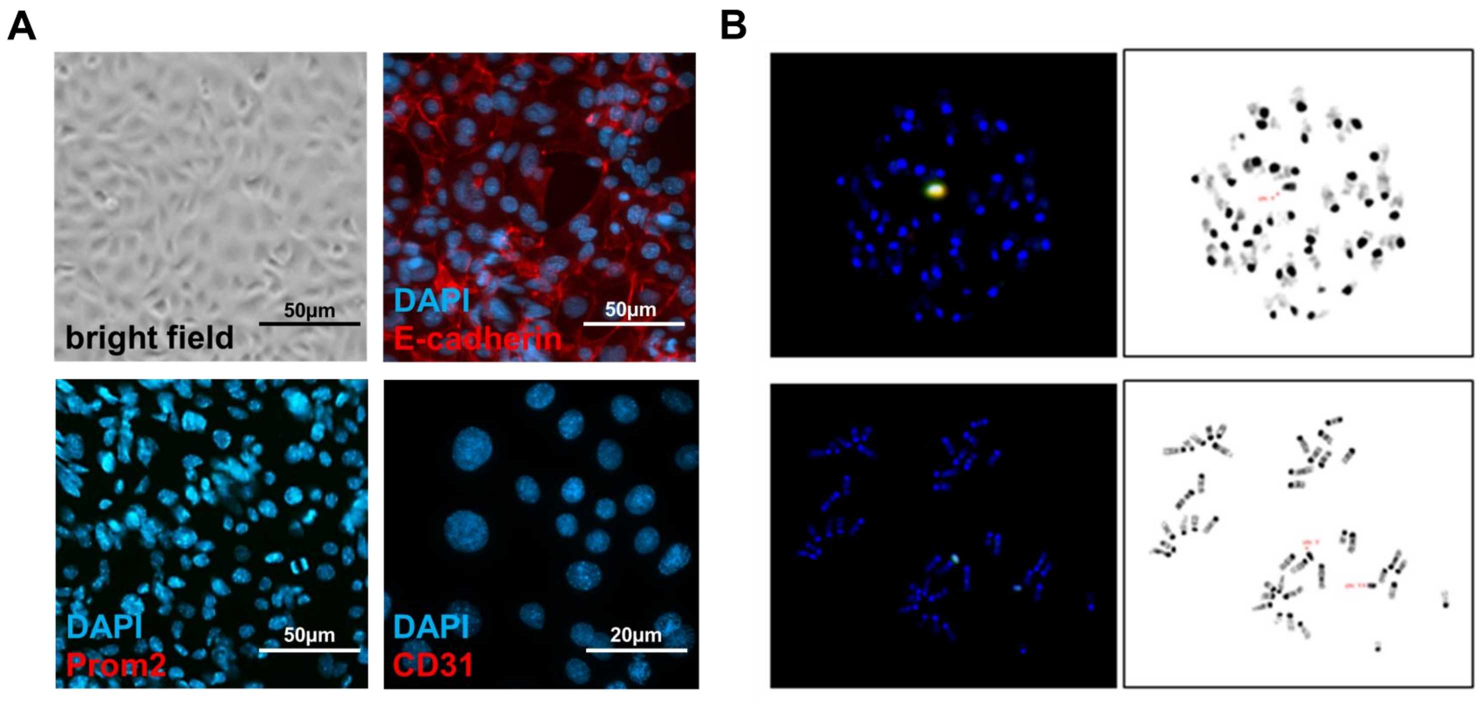

Figure S1: Characterization of TKPTS cells. (**A**) Light microscopic image of TKPTS cells (200×) and immunofluorescence staining with E-cadherin (epithelial cell marker) (200×), Prom2 (distal tubule cell marker) (200×) and CD31 (endothelial cell marker) (630×). (**B**) Giemsa staining and fluorescence in situ hybridization (FISH) metaphase chromosomes. TKPTS cells contained approximately 42 chromosomes per cell. In most cells, only one Y-chromosome copy could be detected per metaphase; in a subset of cells, there were two Y-chromosome copies. However, the murine Y-chromosome-specific wcp probe clearly revealed a male origin of TKPTS cell
